# In-flow single particle detection of sub-100 micron microplastics

**DOI:** 10.1039/d5ra04700e

**Published:** 2025-09-11

**Authors:** Ernesto III Paruli, Agnès De Lavigne Sainte-Suzanne, Mathieu Debeaumont, Lena Thomas, Remi Courson, Lylian Challier, Maria El Rakwe, Enora Prado

**Affiliations:** a IFREMER, RDT Research and Technological Development F-29280 Plouzané France Enora.Prado@ifremer.fr; b Laboratoire ITODYS/UMR 7086, Université Paris Cité – Faculté des Sciences Paris 75013 France

## Abstract

The pervasive and growing contamination of ecosystems by microplastics (MPs) has emerged as a critical environmental and societal challenge. These synthetic polymer fragments, typically defined as plastic particles smaller than 5 mm, are now recognized not only for their persistence in natural environments but also for their potential to carry adsorbed pollutants and to be ingested by a wide range of organisms, including humans. Of particular concern are MPs in the sub-100 μm range, which are more difficult to isolate and analyze but may exhibit enhanced mobility, reactivity, and bioavailability. The accurate detection, quantification, and chemical characterization of such small MPs are therefore essential for advancing our understanding of their sources, fate, and impacts. However, current analytical approaches—primarily based on filtration, staining, and spectroscopic methods—remain time-consuming and often lack the sensitivity or selectivity required for sub-100 μm particles in complex aqueous matrices. In this study, we present a novel microfluidic strategy for the rapid, in-flow detection and molecular identification of individual MPs in suspension. The method integrates dielectrophoresis (DEP) for the label-free spatial manipulation of particles and Raman microspectroscopy (RM) for their chemical fingerprinting. A custom-fabricated glass microfluidic chip was developed, incorporating electrodes on both the top and bottom surfaces of the main channel to achieve three-dimensional DEP focusing. MPs ranging from 25 to 50 μm in diameter were successfully aligned along the channel's central axis and interrogated in real time using RM. This approach enabled unambiguous, particle-by-particle identification of five widely encountered polymer types: polystyrene (PS), polypropylene (PP), polyethylene (PE), polyvinyl chloride (PVC), and polyethylene terephthalate (PET), both in monodisperse and polydisperse mixtures. Our results demonstrate that DEP/RM coupling offers a powerful and scalable platform for in-flow MPs analysis, combining high spatial resolution and chemical specificity. This proof of concept opens new possibilities for high-throughput and automated detection of MPs in environmental monitoring and water analysis.

## Abstract 2

An innovative microfluidic chip adapted to in-flow particle-by-particle microplastic discrimination.

## Graphical abstract

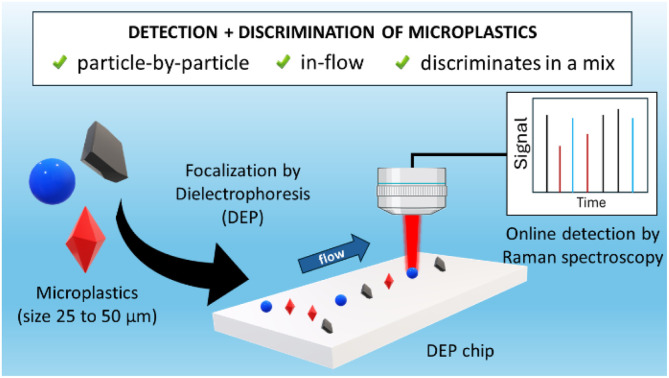

## Introduction

Microplastics (MPs) pollution has emerged as a major environmental concern due to the widespread distribution and persistence of plastic debris in ecosystems. In the oceans alone, over 170 trillion MPs particles were estimated to be afloat as of 2019—a number expected to rise significantly in the coming decades.^1^ MPs have been detected across diverse environments, including marine and freshwater systems,^2–7^ soils,^10,11^ glaciers,^8,9^ and even the atmosphere,^12,13^ exposing a wide range of organisms to potential harm.^14–22^

MPs are typically defined as plastic particles between 1 μm and 5 mm.^23,24^ They can originate from industrial processes (primary MPs) or from the fragmentation of larger plastic waste (secondary MPs). This origin diversity results in highly heterogeneous particles varying in size, shape (*e.g.*, beads, fragments, fibers), and chemical composition.^25,26^ These physical and chemical attributes, especially at smaller sizes (<100 μm), are directly linked to biological risks, such as bioaccumulation, and pollutant adsorption.^27–32^

To address these concerns, reliable MPs detection methods are needed, yet existing techniques face critical limitations. Standard protocols involve multi-step workflows: collection, separation, visual sorting, and spectroscopic identification.^33–36^ While powerful, these methods often suffer from low throughput, require drying or filtration, and become less effective for particles below 60 μm—an increasingly relevant size range. In particular, Fourier-transform infrared spectroscopy (FTIR) is diffraction-limited (∼20 μm) and requires IR-transparent substrates, whereas Raman spectroscopy, although fluorescence-sensitive, allows detection down to a few microns.^33–39^ Both, however, are commonly applied to static, substrate-fixed particles, limiting their relevance for real-time or in-solution analysis.^40–43^

Recent efforts have attempted to overcome these barriers using in-flow detection strategies, particularly with Raman spectroscopy.^44–47^ These setups allow real-time interrogation of particles in suspension, avoiding filtration or drying. However, a major challenge remains: MPs must be precisely aligned with the Raman laser focal spot (∼1–10 μm) for reliable signal acquisition.^48–52^ Without effective particle focusing, the signal-to-noise ratio drops, particle detection becomes inconsistent, and throughput suffers.

Dielectrophoresis (DEP), the movement of particles in non-uniform electric fields, offers a promising solution for contactless, label-free particle focusing within microfluidic channels.^53–55^ Unlike hydrodynamic focusing, DEP enables spatial control based on dielectric properties, regardless of particle shape. Though its application to MPs remains rare—partly due to the complex and irregular nature of environmental MPs^56–60^—recent studies have shown that DEP can successfully concentrate and manipulate biological or synthetic particles in flow.^61–65^

Here, we present a novel in-flow microplastic detection platform based on the coupling of dielectrophoresis and Raman microspectroscopy (DEP/RM). This method enables the chemical identification of individual MPs in suspension by actively focusing them into a Raman interrogation zone. We tested the system on MPs in the 25–50 μm range—an underexplored yet environmentally significant size category^66–69^—and demonstrated its capacity to discriminate five common polymer types (PS, PE, PP, PET, PVC), both in isolation and in complex mixtures. Our results highlight the potential of DEP/RM as a more streamlined, substrate-free, and scalable alternative to conventional MPs detection techniques.

## Results and discussion

### Focusing of microplastics *via* DEP

**Fig. 1:**
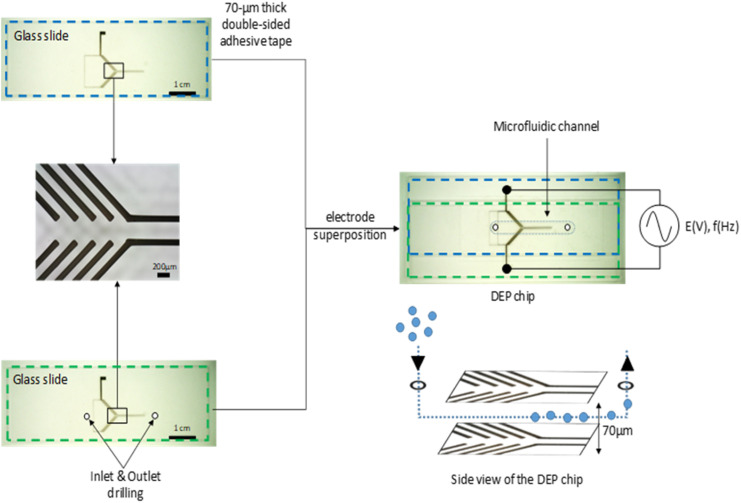
Schematic representation of the DEP chip assembly. Electrodes were fabricated by printing silver conductive ink onto two glass slides (bottom and top slides outlined in blue and green, respectively). The slides were assembled using a 70 μm-thick double-sided adhesive tape, pre-patterned to define a central microfluidic channel.

The DEP chip was the central device for the in-flow detection of microplastics (MPs), as it served as the site for three key processes: (1) in-flow sampling, (2) MPs focusing *via* DEP, and (3) MPs detection through Raman microspectroscopy (RM). Its design was therefore essential for this study. The microchannel was defined by the space delimited laterally by a double-sided adhesive tape sandwiched between two slides of glass. The adhesive tape had a thickness of 70 μm, which established the height of the channel. This height accommodated MPs in the 25–50 μm range while preventing vertical stacking (*i.e.*, particles stacked atop one another) (Fig. 1). The total channel volume was approximately 6 μL, allowing for microsampling of MPs suspensions at any given moment.

**Fig. 2:**
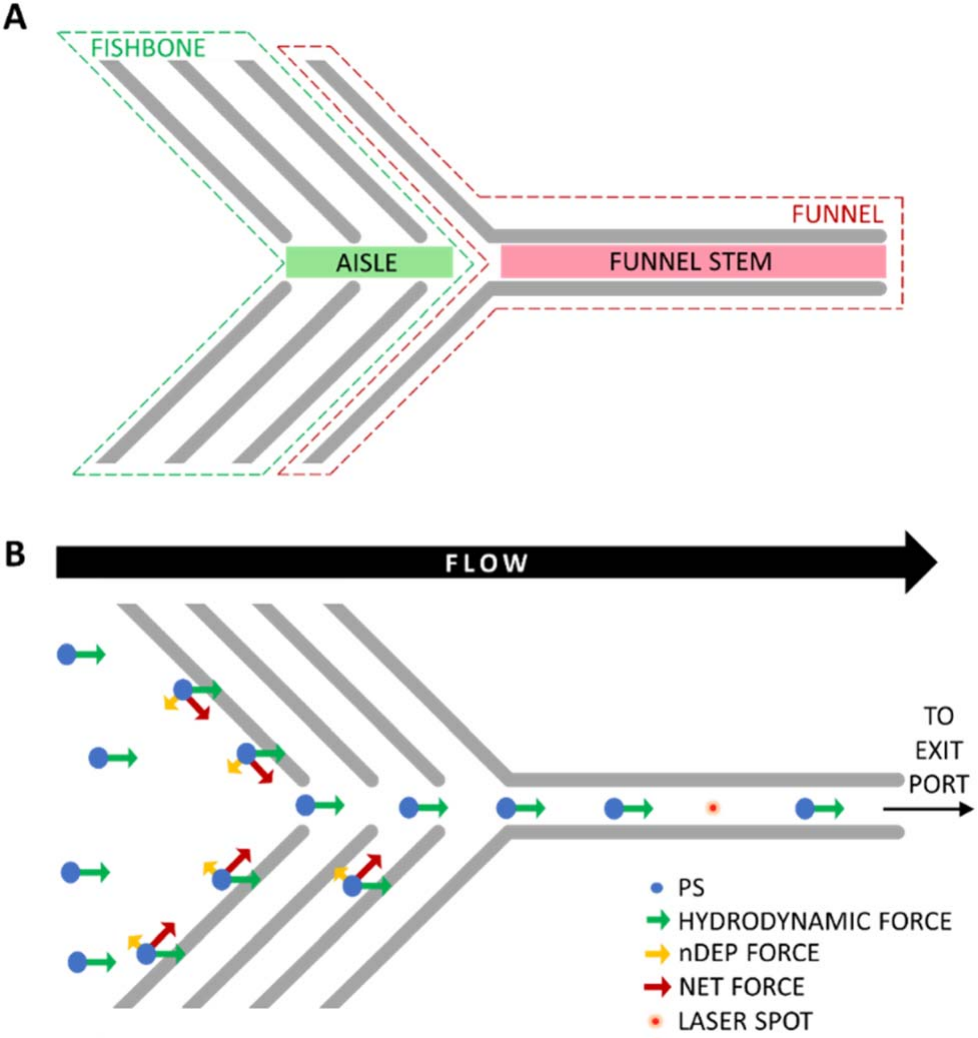
Scheme of (A) the fishbone-and-funnel microelectrode pattern within the microchannel, and (B) the interplay between hydrodynamic drag and negative DEP (nDEP) forces acting on polystyrene (PS) microbeads (Not to scale).

The microelectrodes embedded in the device followed a “fishbone-and-funnel” motif (Fig. 2A), which was key to achieving effective spatial focusing of MPs by aligning hydrodynamic and DEP forces. Upon entering the chip through the inlet, the MPs suspension moved under laminar flow, and the hydrodynamic drag carried the particles forward along a linear path (Fig. 2B). The electrode motif was patterned on both the top and bottom surfaces of the channel, resulting in DEP forces that acted from above and below the particle as it entered the region near the electrodes.

**Fig. 3:**
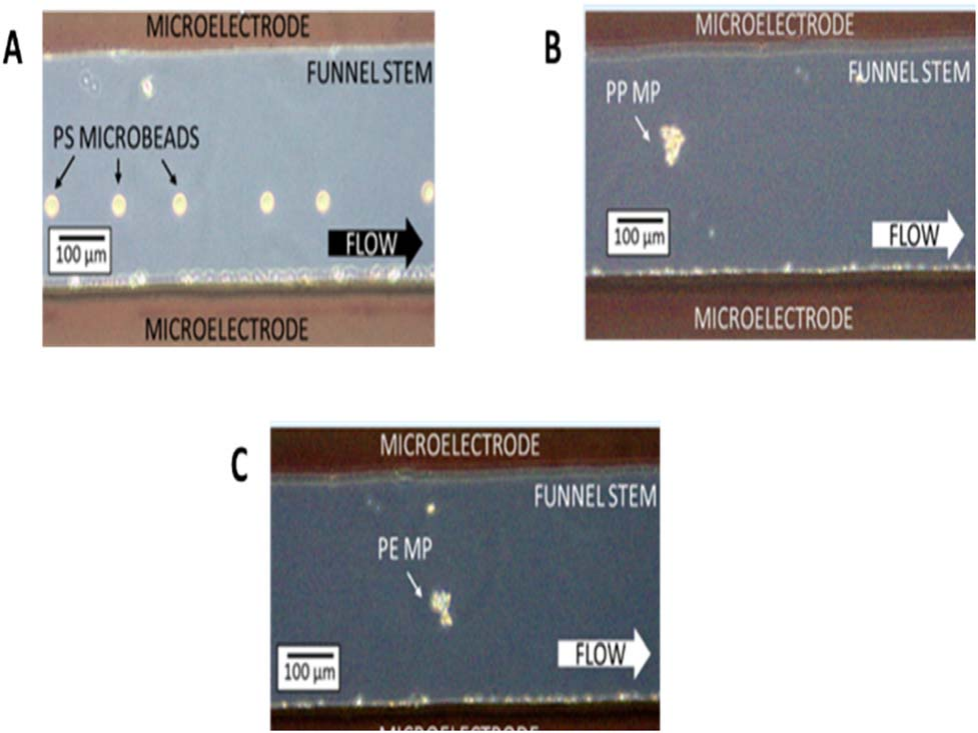
Microscopy images showing the focusing of (A) polystyrene (PS) microbeads, (B) a polypropylene (PP) microplastic, and (C) a polyethylene (PE) microplastic within the stem of the funnel-shaped region of the microchannel.

As the model system, 30 μm-diameter polystyrene (PS) microbeads with spherical geometry were used. These particles exhibited negative DEP (nDEP) at frequencies above 10 kHz, as reported in previous studies.^55,70,71^ As a result, they were repelled from the electrode surfaces. This repulsion was balanced by the hydrodynamic drag, generating a net force that directed the PS microbeads toward the central “aisle” of the fishbone pattern (Fig. 3). Subsequent fishbone electrode pairs served as guiding checkpoints or “safety nets,” further correcting the trajectory of any off-center particles and maintaining their confinement within the central zone.

The funnel-shaped electrode section at the end of the motif acted as the final focusing element. Here, the PS particles entered a 300 μm-wide channel section (the funnel stem), where their motion was sustained by hydrodynamic drag, and lateral confinement was enforced by nDEP. This directed the particles into a narrow path through the focal region of the Raman laser, positioned strategically along the stem for detection. Once the particles passed through the laser focus, they exited the channel *via* the outlet port.

### Focalization of irregular and anisotropic MPs *via* DEP

Unlike PS microbeads, the PP and PE microplastics generated through in-house cryogenic grinding exhibit irregular edges and fall into two morphological categories: (1) three-dimensional (3D) MPs, which are roughly spherical or ellipsoidal, and (2) two-dimensional (2D) MPs, which are disk-like or flat ellipsoids. These shapes reflect the morphology of many PP and PE MPs found in the environment. MPs in the 25–50 μm size range were isolated through serial sieving and validated using FlowCam imaging.

Within the microfluidic channel, when the electric field was applied, both 3D and 2D MPs were repelled from the microelectrode surfaces in close proximity. This behavior was observed to be most efficient in the frequency range of 1–15 MHz. Detailed tracking of MPs motion during DEP focusing revealed distinct behaviors for 3D and 2D particles. 3D MPs demonstrated continuous rotation around their own axis while traversing the fishbone and funnel regions, driven by the interaction between DEP forces and hydrodynamic drag. At the microscopic resolution used in this study, the DEP behavior of these 3D MPs was functionally identical to that of the spherical PS reference particles. In contrast, 2D MPs were subject to both DEP forces and torque. The torque induced a rotation that aligned the longest axis of the particle with the electric field lines—perpendicular to the flow direction. Subsequently, hydrodynamic drag reoriented the flat surface of the particle to align with the direction of fluid flow.

**Fig. 4:**
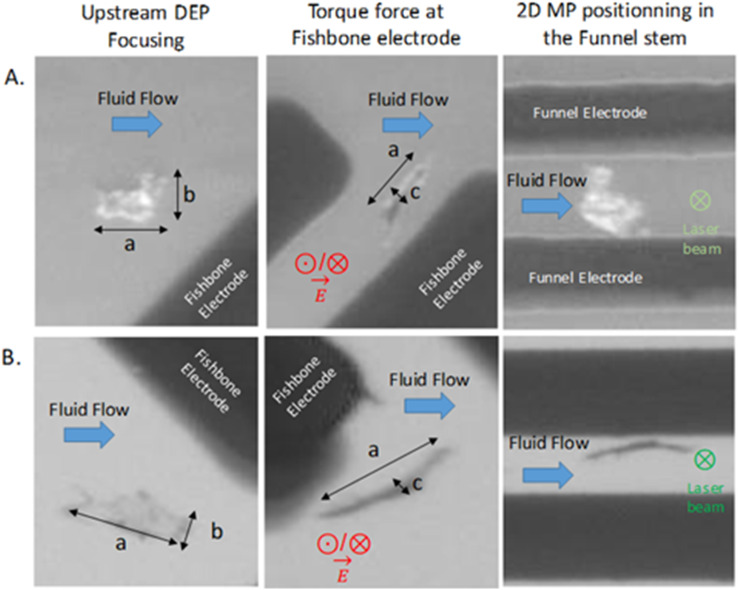
Two 2D particles, A and B, observed in the DEP chip: upstream of the DEP focusing zone, in the fishbone region, and in the funnel stem. Particle dimensions follow the convention *a* (length) > *b* (width) > *c* (thickness). The electric field and the laser beam are oriented perpendicular to the image plane and are indicated by ı (vector pointing out of the plane) and ⊗ (vector pointing into the plane), respectively.

This behavior is illustrated in Fig. 4, which presents two 2D MPs (labeled A and B) with dimensional hierarchy *a* (length) > *b* (width) > *c* (thickness). Upon reaching the fishbone electrode zone, both particles were observed with their smallest dimension (c) oriented toward the viewer, indicating that their largest axes had aligned with the electric field. However, instead of the expected alignment of the longest axis (a) with the field lines, drag forces prevailed, causing the MPs to stabilize with their intermediate axis (b) oriented along the field. This dynamic interplay between torque and drag leads to varying orientation outcomes depending on the local force balance. In the funnel stem, particle B retained the same orientation as in the fishbone region. Particle A, on the other hand, underwent an additional torque-driven reorientation, eventually rotating to face the laser beam directly. This suggests that particle A reached a stable position near the mid-height of the channel, where the electric field gradient is minimized. The contrasting behaviors observed in particles A and B underscore the complex equilibrium states resulting from DEP, torque, and drag forces acting simultaneously on irregularly shaped MPs.

Two MPs focusing issues were observed in the chip. The first stems from excessive MPs speed due to an imbalance between DEP and drag forces, requiring precise flow rates for sufficient Raman analysis time and optimized voltage/frequency settings to ensure reliable MPs guidance for MPs whatever their material. In the following section, a methodology to optimize these experimental conditions was proposed. The second failure involves MPs accumulation at the funnel stem inlet, causing clogging. Both issues were resolved through electrode redesign: the number of fishbone electrode was increased (3 to 6) leading to smoother upstream deflection, while adaptive-curved funnel entries eliminated clogging. These design iterations were accelerated thanks to high-resolution digital printing technology, enabling rapid prototyping within 24 hours.

### DEP and MPs mean speed optimization

Following the focusing effect, the next critical parameter to optimize was the mean speed of MPs as they approached the Raman laser spot. It was necessary to adjust the MPs flow speed to match the acquisition time of the Raman spectrometer and thus obtain a meaningful spectrum from each individual particle. The optimization process was conducted on three types of MPs: 30 μm PS microbeads, and irregular fragments of PP and PE.

The optimization was carried out by varying two key experimental parameters: the flow rate (2, 3, 4, 5, and 6 μL min^−1^) and the electric field frequency (5, 10, and 15 MHz). The lower flow rate limit was set to 2 μL min^−1^, as values below this led to significant particle aggregation near the inlet, which hindered flow. The upper limit was 6 μL min^−1^, beyond which particles moved too quickly to be reliably detected by the Raman beam. The frequency range was selected based on known DEP responses: spherical particles between 10 and 100 μm typically exhibit negative DEP in the MHz range,^55,70,71^ while positive DEP occurs in the kHz range.

Although the dielectrophoretic behavior of irregularly shaped MPs is difficult to predict, the dipole (spherical) approximation was used as a starting point to identify suitable field frequencies for a broad population of particles. Furthermore, the applied frequency also influences the magnitude of the DEP force and was therefore explored systematically.

A full factorial experiment using all combinations of the flow rate and frequency values would have required 60 separate tests for each MPs type. To reduce this workload, a Design of Experiments (DOE) approach was employed. DOE enables the identification of key parameter interactions and effects using a minimal number of well-structured experiments. Using JMP software, an optimal design was generated, reducing the required experiments to 27 per MPs type.

**Fig. 5:**
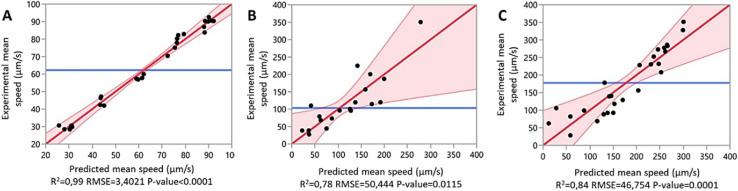
Experimental mean speed *versus* predicted mean speed for (A) PS, (B) PP and (C) PE.

The mean particle speed for each test was calculated from recorded videos using the TrackMate plugin in Fiji. The resulting speeds, along with the corresponding experimental parameters, were entered into JMP to generate predictive models for PS, PP, and PE (Fig. 5) (example values for PS are shown in Table S1). Additional morphological parameters—aspect ratio, area, circularity, perimeter, and short/long radius—were also included in the model.

Each data point in the model represents an experimentally measured mean speed, while the shaded red area represents the 95% confidence interval of the predicted values. The strong agreement between experimental and predicted values, reflected by high *R*^2^ values of 0.99 (PS), 0.78 (PP), and 0.84 (PE), confirmed the robustness of the model.

**Fig. 6:**
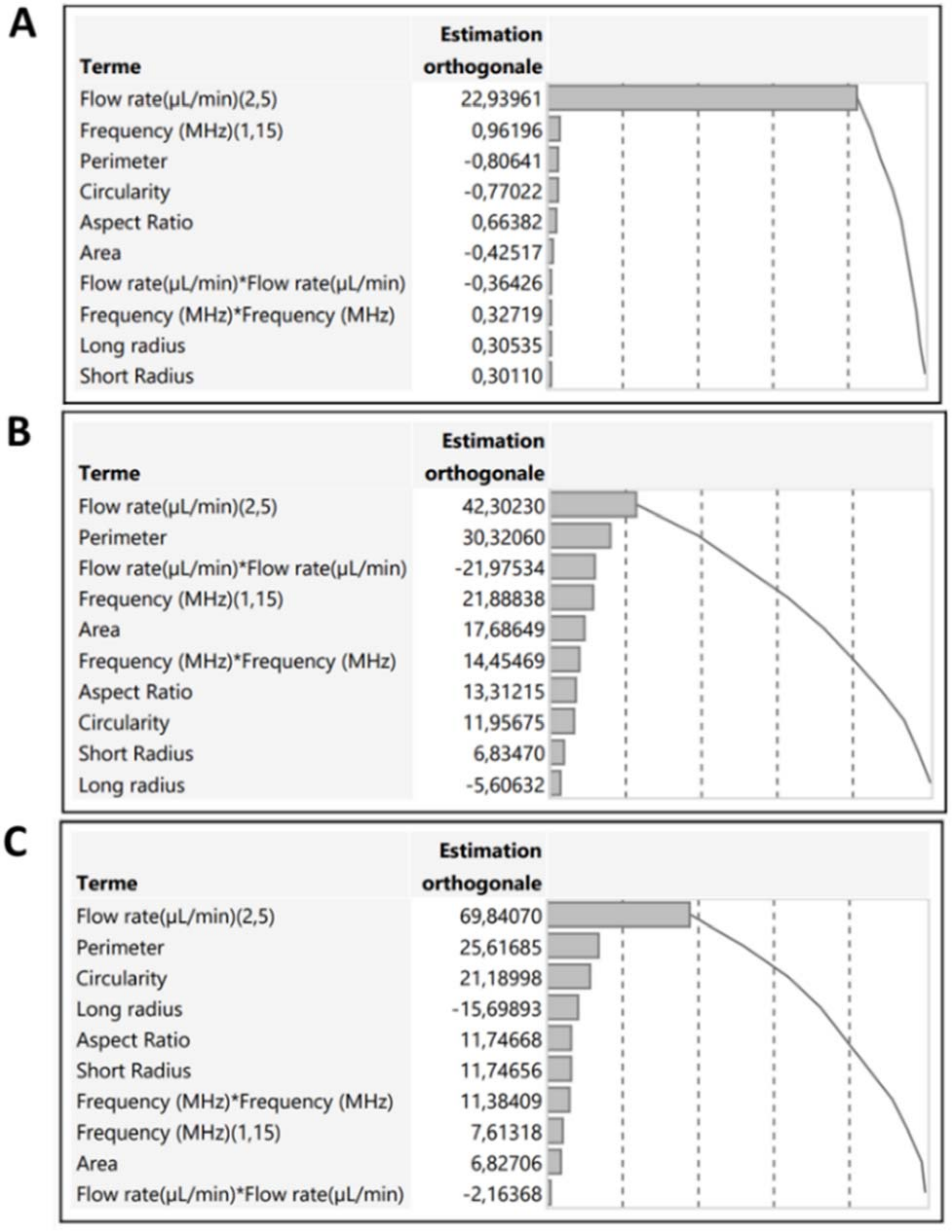
Pareto diagrams for (A) PS, (B) PP and (C) PE.

Fig. 6 presents Pareto charts summarizing the impact of each studied parameter on MPs mean speed. Factors with effect bars crossing the significance threshold (*p* > 0.05) were considered statistically insignificant.

The results show that flow rate was the only significant factor influencing MPs speed across all three polymer types. As expected, the correlation was directly proportional: increasing the flow rate resulted in faster particle transport. Although low flow rates yielded better spectral acquisition per particle, they were impractical for PP due to low throughput. As a result, the minimum usable flow rate was set at 3 μL min^−1^ for all MPs.

In contrast, frequency had no statistically significant impact on mean speed. Therefore, a fixed frequency of 5 MHz was selected for subsequent experiments. Under these optimal conditions (flow rate = 3 μL min^−1^, frequency = 5 MHz), the predicted mean speeds for PS, PP, and PE were 43, 75, and 108 μm s^−1^, respectively.

At these speeds, DEP focusing achieved near-perfect efficiencies: 100% for PS and PE (*n* = 100 each), and 87% for PP (*n* = 100). In comparison, under passive flow conditions (no DEP), only 6–9% of particles passed through the detection zone (see Table S2), highlighting the crucial role of DEP in guiding MPs effectively into the Raman beam.

### MPs detection *via* DEP/RM coupling

DEP was instrumental in spatially focusing MPs so that they could be accurately delivered to the focal spot of the laser beam projected into the microchannel from an overhead Raman microspectrometer. For context, the theoretical diameter of the focal spot of the 633 nm laser used in this study was estimated at approximately 1.54 μm, according to Abbe's formula:
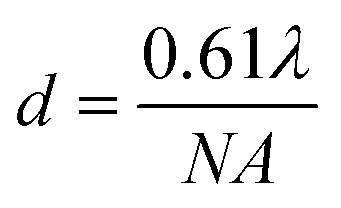
where *d* is the focal spot diameter, *λ* is the laser wavelength (633 nm), and *NA* is the numerical aperture of the objective lens, set at 0.25. Given the extremely small interrogation volume, tightly confining the MPs within the focal region was essential to enable detection by RM.

MPs detection in this study was achieved through successive acquisition of individual Raman spectra over time at a fixed location along the funnel stem—an approach referred to as dynamic RM. This method allowed for real-time, particle-by-particle chemical identification. The acquisition time was tuned based on the optimal mean speeds previously established for each polymer to ensure sufficient interaction time between the laser and each MPs.

For PS microbeads, the optimal speed was 43 μm s^−1^. Given the bead diameter of 30 μm, each particle required approximately 0.70 s to traverse the laser spot. With acquisition times set to this duration, each spectrum captured ideally corresponded to a single microbead, provided that the inter-particle spacing exceeded the particle size (as confirmed in Fig. 3). This ensured that each spectrum contained a clearly identifiable PS signature rather than background noise.

**Fig. 7:**
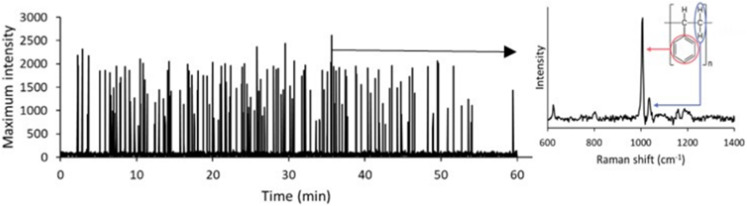
Raman signal of PS monitored over time as the maximum intensity between 980 and 1020 cm^−1^.

Fig. 7 illustrates a representative Raman spectrum of a PS microbead, showing a prominent peak at 1000 cm^−1^ corresponding to the phenyl ring breathing mode. This peak was consistently the most intense in the PS spectrum and was therefore monitored as the defining “signal” for PS detection. By acquiring successive spectra every 0.7 seconds and tracking the 980–1020 cm^−1^ region, a time-resolved signal trace was produced. Each peak in the intensity-*vs*-time plot corresponds to a detected PS microbead, demonstrating successful real-time single-particle detection *via* DEP/RM coupling.

The detection approach for PP and PE was slightly adapted due to their broader size range (25–50 μm). An average length of 37.5 μm was used to define an effective acquisition window. Based on their mean flow speeds (75 μm s^−1^ for PP and 108 μm s^−1^ for PE), optimal acquisition times were calculated as 0.5 s and 0.3 s, respectively. These acquisition times captured sufficient spectral data as particles passed through the laser spot.

In practice, when the leading edge of a 50 μm MPs entered the Raman spot, the initial spectrum covered about 37.5 μm of the particle, while the remaining 12.5 μm was captured in a subsequent spectrum. The second spectrum, although less intense, still contributed to MPs identification. Characteristic Raman regions were monitored—805–855 cm^−1^ for PP (CH_3_ rocking and C–C stretching peaks at 814 and 847 cm^−1^) and 1277–1317 cm^−1^ for PE (CH_2_ twisting mode at 1297 cm^−1^).

**Fig. 8:**
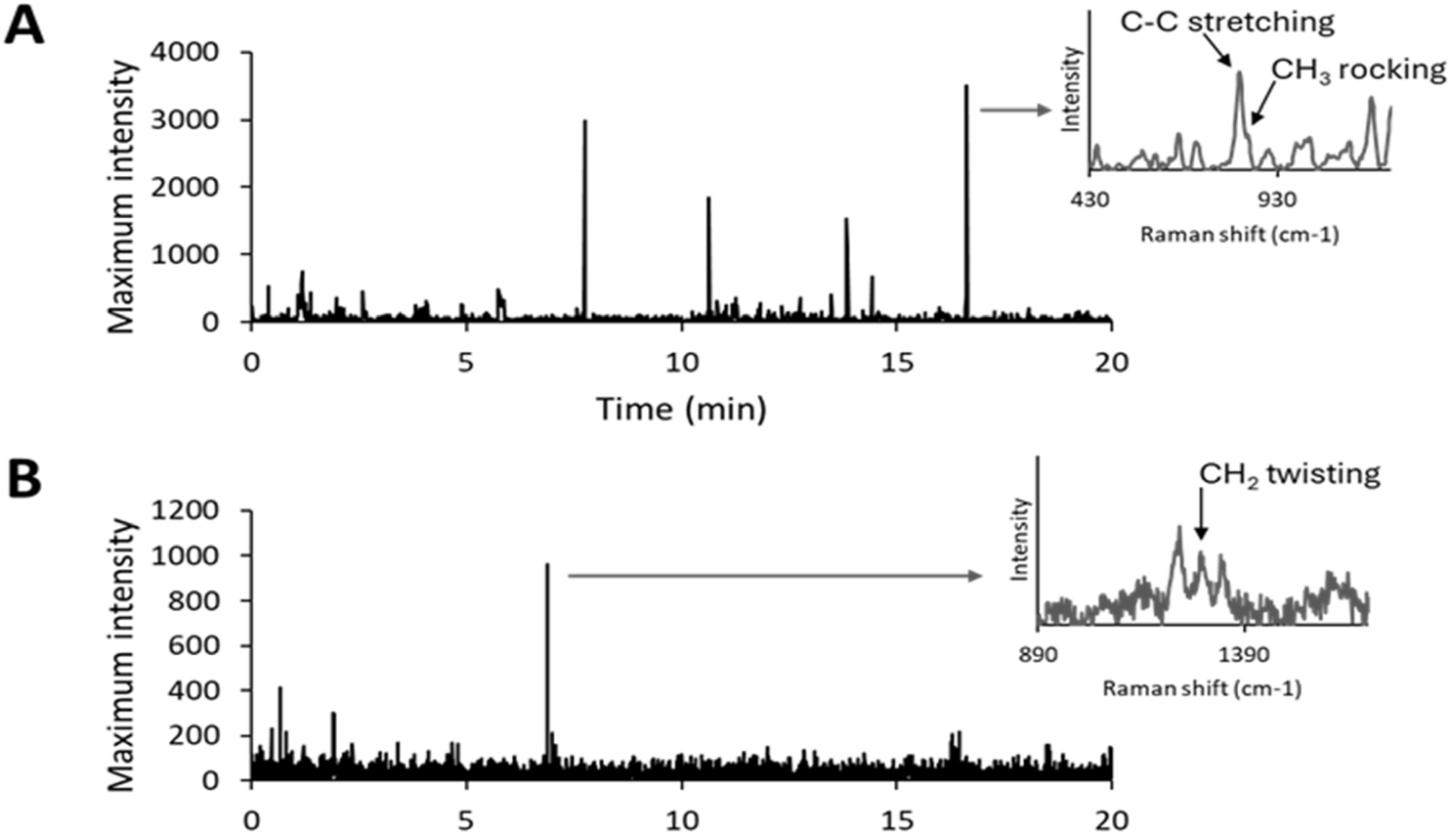
Raman signal of (A) PP and (B) PE monitored over time as the maximum intensity between 805 and 855 cm^−1^ and between 1277 and 1317 cm^−1^, respectively.

Fig. 8 shows the corresponding intensity-*vs*-time plots for PP and PE, confirming successful detection of both MPs types. Thus, DEP/RM coupling enabled single-particle detection not only of spherical microbeads but also of irregularly shaped polymer fragments in flowing suspension.

### Discrimination of MPs types *via* DEP/RM coupling

The DEP/RM platform was further applied to distinguish between multiple MPs types in mixed suspensions. For this, a unified acquisition time of 0.5 seconds was selected—an intermediate value between the optimal acquisition times previously used for PS, PP, and PE. This acquisition time ensured sufficient interaction for a broad range of particle sizes (25–50 μm), while maintaining reasonable temporal resolution.

During dynamic Raman analysis, the spectral regions corresponding to the characteristic peaks of each polymer were monitored simultaneously: 980–1020 cm^−1^ for PS, 814–847 cm^−1^ for PP, 1277–1317 cm^−1^ for PE.

**Fig. 9:**
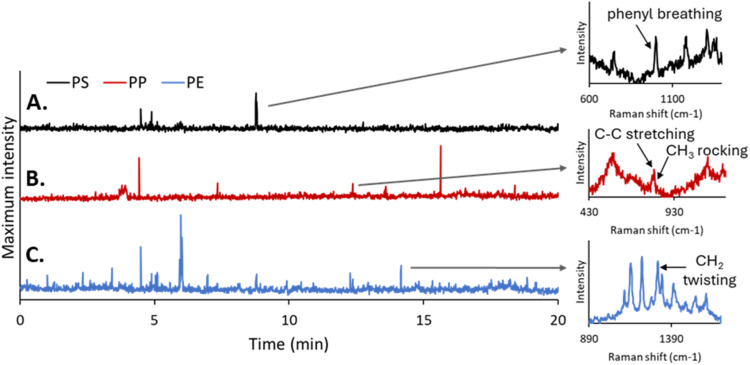
Discrimination of (A) PS, (B) PP and (C) PE by monitoring the maximum intensity in their respective characteristic spectral range of Raman signal (980–1020 cm^−1^; 814–847 cm^−1^; 1277–1317 cm^−1^, respectively) over time.

As shown in Fig. 9, the intensity-*versus*-time plots for each spectral window display peaks at different time points, each corresponding to the detection of a specific MPs type. Monitoring the 980–1020 cm^−1^ region yielded distinct PS signals (Fig. 9A), while shifting the spectral window to 814–847 cm^−1^ revealed peaks attributed to PP (Fig. 9B). Similarly, PE detection was achieved by analyzing the 1277–1317 cm^−1^ window (Fig. 9C). These results confirm the capability of the system to discriminate between individual particles of different polymer types in real-time and under flow conditions.

To further assess the robustness of the approach, the method was extended to a more complex suspension containing five MPs types: PS, PE, PP, PET (polyethylene terephthalate), and PVC (polyvinyl chloride). Particle sizes again ranged from 25 to 50 μm. Two additional spectral windows were used for identification: 670–720 cm^−1^ for PVC (characteristic Cl–C stretching), 1600–1650 cm^−1^ for PET (aromatic ring C=C stretching). As shown in Fig. S3, all five polymers were successfully detected during time-resolved analysis. Reference Raman spectra for each MPs type are provided in Fig. S1.

This experiment demonstrates that the DEP/RM platform is not only capable of single-particle detection but also enables the simultaneous identification and discrimination of multiple polymer types in complex mixtures. The ability to resolve these types in dynamic, aqueous systems is critical for practical applications such as environmental monitoring or on-site analysis of microplastic contamination.

These findings underline the practical advantages of the DEP/RM approach over conventional MPs detection methods. Traditional workflows often involve static analysis of dried samples under the microscope or labor-intensive filtration steps followed by *ex situ* spectroscopic identification.^33^ While techniques such as FTIR imaging or Raman mapping provide chemical specificity, they suffer from limited throughput and require particle immobilization on substrates—making them poorly suited for real-time or in-solution analysis.^37^ Moreover, size thresholds inherent to FTIR (>20 μm) and optical microscopy (>60 μm) often exclude smaller MPs from detection pipelines.^35,39^

In contrast, the DEP/RM system enables direct analysis of MPs in suspension, under flow, and at the single-particle level. The active focusing provided by dielectrophoresis improves alignment and ensures sufficient interaction time with the Raman laser, even for irregularly shaped particles. This overcomes a major challenge faced by in-flow Raman approaches that rely solely on hydrodynamic focusing, which often fails to maintain consistent spatial overlap between particles and the laser focal volume.^50^ The system's ability to resolve multiple MPs types in dynamic conditions, without labeling, filtration, or drying, opens new perspectives for rapid, field-adaptable microplastic analysis—bridging the gap between high-resolution laboratory tools and real-world monitoring needs.

### Toward a quantitative analysis

The results obtained so far demonstrate that DEP/RM coupling enables particle-by-particle detection of MPs, suggesting its potential for quantification. In theory, each intensity peak in the Raman intensity-*versus*-time plot corresponds to a single MPs passing through the laser spot, making it possible to count MPs directly.

To test this hypothesis, PS microbeads at various concentrations were introduced into the DEP/RM system. The number of intensity peaks was recorded and translated into estimated concentrations. These were compared to reference concentrations obtained using FlowCam, the standard particle-counting tool in this study. As shown in Fig. S4, the concentrations measured by both methods were generally in the same order of magnitude, although not perfectly aligned.

The discrepancies may be attributed to the heterogeneous nature of MPs suspensions, which are not true solutions. Non-uniform particle distribution likely introduced variation during intermediate steps (*e.g.*, aliquoting, dilution, or injection into the DEP chip). Additionally, incomplete delivery of particles into the microchannel may have further skewed the quantification.

In the case of PP and PE fragments, the particle counts obtained *via* DEP/RM were significantly lower than those from FlowCam (data not shown). Microscopic examination revealed MPs trapped along the channel walls and inlet region, indicating losses prior to detection.

To address this issue, one strategy tested was to increase the concentration of Triton X-100 in the suspending medium, aiming to reduce surface adhesion of MPs. However, this yielded limited improvement. Above 3% Triton, bubble formation became problematic and disrupted flow stability. As Triton X-100 was the only available surfactant during the experiments, further investigation into alternative non-ionic surfactants compatible with DEP could enable higher concentrations and better dispersal without bubble formation.

Another optimization target lies in improving the alignment of inlet and outlet holes between the adhesive layer and the glass slides of the chip. In the current design, the adhesive holes were intentionally larger than those in the glass to simplify assembly. Refining these dimensions could enhance flow continuity and increase particle injection efficiency.

A more radical improvement involves eliminating the current sampling tube setup entirely. With modern 3D printing and soft-lithography, it would be feasible to design conical PDMS inlet ports tailored to accept standard pipette tips. These could be bonded to the chip *via* plasma treatment or adhesive. This approach would enable direct, bubble-free loading of the MP suspension into the device—reducing the need for stirring and avoiding tubing altogether.

Despite the current limitations, these experiments represent a promising foundation for the development of a truly quantitative in-flow microplastic analysis platform. With further refinement in chip design, flow handling, and calibration, DEP/RM coupling has the potential to evolve into a robust and reliable tool for both qualitative and quantitative MPs monitoring.

## Conclusions

This study has demonstrated the feasibility of coupling dielectrophoresis (DEP) with Raman microspectroscopy (RM) to achieve real-time, in-flow detection and chemical identification of individual microplastics (MPs) in the 25–50 μm size range. Importantly, this method proved effective not only for spherical, monodisperse particles (PS) but also for irregularly shaped fragments (PP, PE, PET, PVC), highlighting its relevance for real-world environmental samples.

The key innovation lies in the use of a custom microfluidic chip with a fishbone-and-funnel electrode design, enabling precise focusing of MPs *via* negative DEP. This active control of particle trajectories ensures consistent passage through the Raman interrogation zone, thus enabling dynamic spectral acquisition without the need for filtration or surface deposition.

Compared to conventional multi-step protocols—which often involve filtration, drying, and static particle analysis—this platform offers a streamlined, substrate-free approach with real-time capabilities and non-destructive analysis. The ability to discriminate multiple MPs types (PS, PE, PP, PET, PVC) in mixed suspensions further strengthens its value for complex sample analysis.

While initial quantification tests showed that DEP/RM can approximate MPs concentrations, current limitations such as particle adhesion, flow irregularities, and channel losses highlight the need for further optimization. Engineering efforts to improve inlet geometry, explore alternative surfactants, and eliminate tubing are ongoing, with the goal of enhancing reproducibility and detection efficiency.

Beyond its standalone potential, this platform could also be integrated with complementary detection systems. For instance, coupling DEP/RM with machine learning-based signal processing, fluorescence-based triggers, or in-line flow cytometry could further enhance sensitivity, automation, and selectivity—especially in complex or turbid matrices. Such multimodal configurations may enable hybrid screening workflows combining physical sorting and chemical fingerprinting.

Looking forward, applying this technology to real-world samples will require addressing several challenges: miniaturization for portability, robustness under variable field conditions, pre-filtration or pre-enrichment modules to handle environmental matrices, and intelligent software for signal discrimination and automated classification. Efforts in these directions are essential to transition this approach from lab-based proof-of-concept to a deployable, field-ready device.

In summary, the DEP/RM platform represents a promising advance in the detection of sub-100 μm MPs, bridging a critical gap between high-resolution spectroscopy and in-flow analysis. With further refinement and system integration, it holds strong potential for both qualitative and quantitative monitoring of microplastics in environmental, industrial, and potentially biomedical contexts.

## Experimental

### Materials

Monodisperse PS microbeads in aqueous suspension (diameter = 30 μm, std dev <0.4 μm, coeff var <1%, concentration ≈ 1.3 × 10^6^ microbeads per mL, analytical standard) was obtained from Sigma-Aldrich, Switzerland. Triton X-100 was acquired from Chimie-Plus Laboratoires, France. 96% ethanol was purchased from VWR, Belgium.

### Fabrication of PP and PE MPs

Red file fasteners, blue water bottle caps, piece of gutter and water bottle served as raw materials for the fabrication of polypropylene (PP), polyethylene (PE), polyvinyl chloride (PVC), and polyethylene terephthalate (PET) MPs, respectively (chemical identity confirmed by Raman spectroscopy in static mode (HR800, Horiba scientific, Japan) (Fig. S1)). PP, PE, PVC and PET MPs in the size range of 25–50 μm were fabricated by (1) cryogenic grinding of raw materials and (2) fraction separation by sieving.

### Cryogenic grinding of raw materials

For each MPs type, the raw material was cut into small pieces of a few millimeters and distributed into four 2 mL cryotubes. The cryotubes, along with two 50 mL inox grinding jars and four 1.5 cm inox grinding balls, were then cooled in liquid nitrogen for 10 minutes. Afterwards, each jar received the contents of 2 cryotubes and 2 balls. The jars were then fixed into a MM 400 mixer mill (Retsch, France), which in turn operated for 5 minutes at 30 Hz. Finally, the MPs inside the jars were stored in a container until they attained room temperature.

### Fraction separation by sieving

A mixture of 1.5 g of MPs in 100 mL ethanol was ultrasonicated in a beaker for 2 minutes. Meanwhile, a vacuum filtration was set up, an inox test sieve (mesh size: 1000 μm) was attached to the top of the Büchner funnel. The mixture was then poured into the sieve, which was thereafter rinsed generously with ethanol. The sieve was detached and its contents placed in a 500 mL Schott reagent bottle, designated as Fraction 1 (F1). The filtrate was then recovered and sieved for two more times and the sieve contents were deposited in the same F1 bottle.

The entire process was repeated for the resulting filtrate, each time with a sieve of lower mesh size (350, 100, 50 and 25 μm) and the contents stored in separate bottles (F2, F3, F4, F5, F6). The content of the 25 μm sieve was called Fraction 5 (F5) and was composed of MPs between 25 and 50 μm. The F5 MPs were allowed to settle at the bottom of their bottle overnight, then excess supernatant was removed. The MPs in ethanol was then stored in a 50 mL conical tube until use.

### Particle counting *via* FlowCam

The number of MPs in a suspension was determined through the use of the flow imaging microscopy instrument FlowCam 5000 (Yokogawa Fluid Imaging Technologies, USA) equipped with 4*x* objective and a flowcell of 300 μm. After rinsing the flow system with filtered 0.5% w/w Triton/water, the cone was filled with an aliquot of the suspension. Once the program was launched, around 1000 μL of the aliquot was automatically aspirated through the flow cell and analyzed in triplicate. The analysis yielded the number of particles, sample volume analyzed and several morphological information such as mean length and mean width. An example of results for PP, PE, PVC and PET are shown in the SI (Fig. S2). MPs concentration was then calculated by dividing the number of particles by the volume analyzed.

### Sample preparation

For both DEP and DEP/RM experiments, PS suspension at a concentration of 10 000 MPs/mL was prepared by diluting commercially supplied PS microbeads in Milli-Q water containing 0.5% w/w Triton X-100. Suspensions of PP, PE, PVC, and PET were prepared by first removing the ethanol from their respective stock solutions, followed by resuspension in 0.5% w/w Triton X-100 in Milli-Q water. Two types of mixed suspensions were used: the first consisted of PS, PE, and PP microplastics, each representing one-third of the total concentration, yielding a final concentration of 10 000 MPs per mL. The second mixture included all five polymer types (PS, PE, PP, PET, and PVC) with a total final concentration of 10 000 MPs per mL.

### Fabrication of the DEP chip

Silver conductive ink was deposited on one side of a 7.5 × 2.5 × 0.1 cm^3^ glass slide using a 30 μm internal diameter glass micropipette (WPI, USA) and the NAZCA direct-writing system (HUMMINK, France), to fabricate the electrode patterns shown in Fig. 1. The same procedure was repeated on a second glass slide that had been previously drilled with a diamond bit to create circular inlet and outlet holes (diameter ≈ 1 mm) at both ends. The two slides were then assembled with the electrode-patterned sides aligned and facing each other, separated by a 70 μm-thick double-sided adhesive spacer (Montex DX2, X-Film, Germany), which had been pre-patterned using a plotter to define a central microfluidic channel. The resulting device formed the DEP chip, with a channel incorporating the electrode structures and connecting the inlet and outlet ports.

This approach enables the fabrication of a cost-effective (€150–200) dielectrophoresis (DEP) chip using off-the-shelf components and without requiring cleanroom facilities, making it particularly suitable for academic laboratories. The reversible, tape-based assembly facilitates electrode aging studies and the replacement of 3D-printed fluidic connectors. Optimized tubing lengths reduce dead volumes. Elevated voltages (10 V peak-to-peak) can accelerate electrode delamination; however, UV/ozone pretreatment of the glass substrates prior to ink deposition significantly enhances electrode adhesion. Operation at low frequencies (<1 kHz) or under DC bias is discouraged due to the electrochemical reactivity of silver, which can lead to water electrolysis and/or electrode dissolution. No signs of adhesive aging, such as UV-induced yellowing, cracking, or drying, were observed for the double-sided tape. Cleaning with non-ionic Triton detergent and regular tubing replacement effectively prevent cross-contamination. The adhesives remain intact as long as ethanol or acetone are not circulated through the channel. Once operating and fabrication conditions are optimized, the chips exhibit a functional lifetime exceeding 30 hours without DEP failure or leakage.

### Design of experiment (DOE)

For the optimization of MPs mean speed, two parameters were investigated: the flow rate at 5 different levels (2, 3, 4, 5, 6 μL min^−1^) and the electric field frequency at 4 levels (1, 5, 10, 15 MHz) at a fixed voltage (20 V). An optimal experimental design consisting of 27 experiments (Table S1) was determined using the software JMP.^75^ The data collected from these would later be used to generate prediction profiles correlating the parameters to the MPs mean speed.

### DEP experiments

A 2 mL MPs suspension in constant magnetic stirring was contained in a 4 mL glass vial. One end of a Tygon tube (inner diameter = 0.38 mm) was immersed in the suspension while the other was connected to the inlet of the DEP chip through a 3D printed holder. Likewise, a second tube was attached to the exit port and to a syringe maneuvered by a syringe-pump. Through the withdrawing action of the syringe, a small volume of the MPs suspension was introduced into the chip at a flow rate set on the NE-1000 syringe-pump (New Era Pump Systems Inc., USA). Meanwhile, the printed electrodes on the inlet and outlet slides of the chip were linked to a DG1022 AC generator (Rigol, France). The AC voltage was set to 20 Vpp and to different frequencies as needed. To monitor particle behavior visually inside the chip, it was fixed on the stage of an Axio Vert. A1 inverted microscope (Carl Zeiss, France). A camera was coupled to the microscope to capture images and videos and send these to an interfaced computer. The PS suspension was introduced into the chip. The flow rate and the frequency were adjusted according to the DOE. Likewise, the DOE was applied separately to PP and to PE.

### Video analysis *via* TrackMate plugin on Fiji

Videos recorded during the optimization of DEP conditions were processed using the Fiji software (see SI-video tracking). Each video was first converted into a stack of grayscale images.^72^ Particle detection was performed using the TrackMate plugin,^73^ with the Thresholding Detector, which identifies particles based on image contrast relative to a defined intensity threshold. Particle trajectories were subsequently tracked using the nearest-neighbor tracker, yielding their mean velocities. In addition to tracking data, morphological parameters such as aspect ratio, area, circularity (equal to 1 for a perfect circle and approaching 0 for highly elongated shapes), perimeter, and the long and short radii were extracted.^74^ The long and short radii correspond to the semi-axes of an ellipse best fitting the particle's contour.

### MPs detection *via* DEP/RM coupling

The DEP chip was positioned on the stage of a LabRAM HR800 Raman microspectrometer (HORIBA Scientific, France). For in-flow detection of MPs, a 20× objective with a numerical aperture (NA) of 0.25 was used, and a 633 nm monochromatic laser served as the excitation source. The stage was adjusted in the *XY* plane to align the laser spot with the trajectory of the MPs. For polystyrene (PS), the *Z*-axis was adjusted to focus the laser on the mid-plane of the microbeads. For polypropylene (PP), polyethylene (PE), and their mixture with PS, the optical focus was set at the midpoint between the top and bottom electrodes.

Upon injection of the MPs suspension into the chip, dynamic Raman spectra were acquired continuously over time, with acquisition times of 0.7 s (PS), 0.5 s (PP) and 0.3 s (PE), and 0.5 s for the mixed suspension. Specific spectral regions containing the characteristic Raman peaks of each polymer were monitored. All spectra were baseline-corrected and analyzed using the LabSpec 6 software (HORIBA Scientific).

## Author contributions

Ernesto III Paruli: writing –original draft, writing – review & editing, visualization, investigation, methodology, validation, formal analysis; Enora Prado: writing – review & editing, investigation, methodology, validation, conceptualization, project administration, supervision, funding acquisition; Maria El Rakwe: writing – review & editing, investigation, methodology, formal analysis, validation, conceptualization, project administration, supervision; Agnès De Lavigne Sainte-Suzanne: investigation, validation; Mathieu Debeaumont: investigation, validation, resources; Léna Thomas: writing – review & editing, investigation, methodology, validation, resources; Rémi Courson: writing – review & editing, investigation, methodology, validation, conceptualization; Lylian Challier: writing – review & editing, investigation, methodology, validation, conceptualization.

## Conflicts of interest

There are no conflicts to declare.

## Supplementary Material

RA-015-D5RA04700E-s001

RA-015-D5RA04700E-s002

## Data Availability

The data supporting this article have been included as part of the SI. Supplementary information: Reference Raman spectra, data from particle counting *via* FlowCam. DOE data, video tracking. See DOI: https://doi.org/10.1039/d5ra04700e.
