# Supplementary material for: In-flow single particle detection of sub-100 micron microplastics
Source: RSC Adv. 2025 Sep 11;15(40):33139–50. doi: 10.1039/d5ra04700e (PMC12424377; doi:10.1039/d5ra04700e)
Supplement: RA-015-D5RA04700E-s001 [file RA-015-D5RA04700E-s001.pdf]

Supplementary Information (SI) for RSC Advances.
This journal is © The Royal Society of Chemistry 2025

SUPPLEMENTARY INFORMATION

IN-FLOW SINGLE PARTICLE DETECTION OF SUB-100 MICRON MICROPLASTICS

Ernesto III Paruli[a], Agnès De Lavigne Sainte-Suzanne[a], Mathieu Debeaumont[a], Lena Thomas[a], Remi Courson[a], Lylian Challier[b], Maria El Rakwe[a], Enora Prado*[a]

[a]IFREMER, RDT Research and Technological Development, F-29280 Plouzané, France

[b]Laboratoire ITODYS/UMR 7086, Université Paris Cité – Faculté des sciences, Paris 75013, France

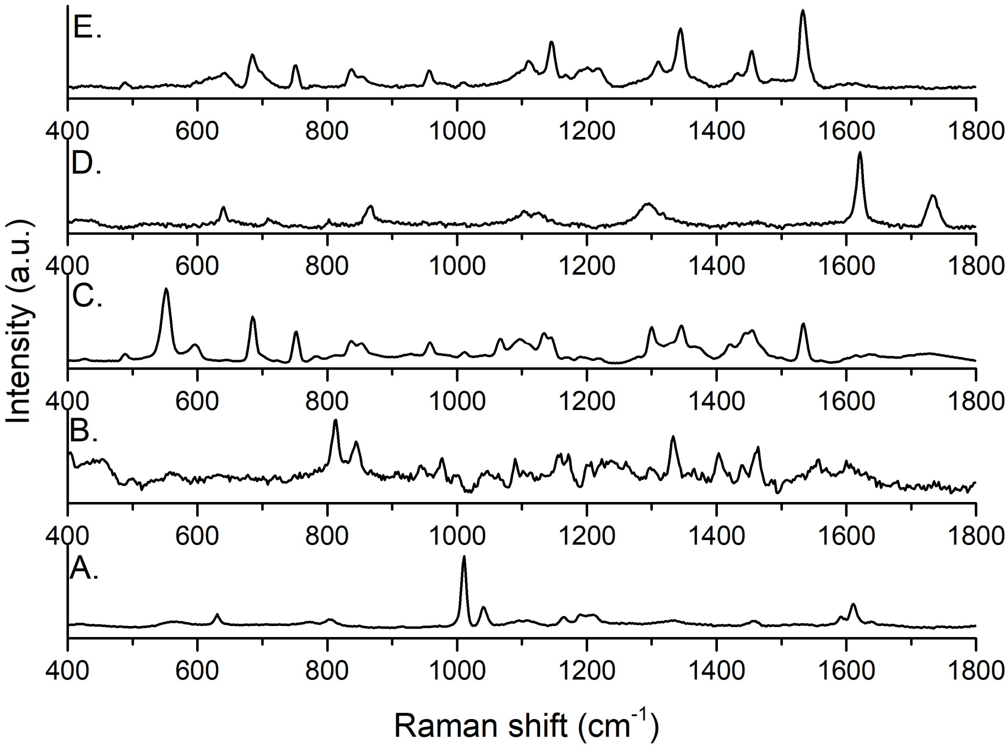

Figure S1. Reference Raman spectra of (A) PS microbeads, (B) PP, (C) PE, (D) PET and (E) PVC fragments acquired in static mode (λ_{exc} = 633 nm; 300 tr/mm; Obj. 20x, Acq. = 30 s) after background subtraction.

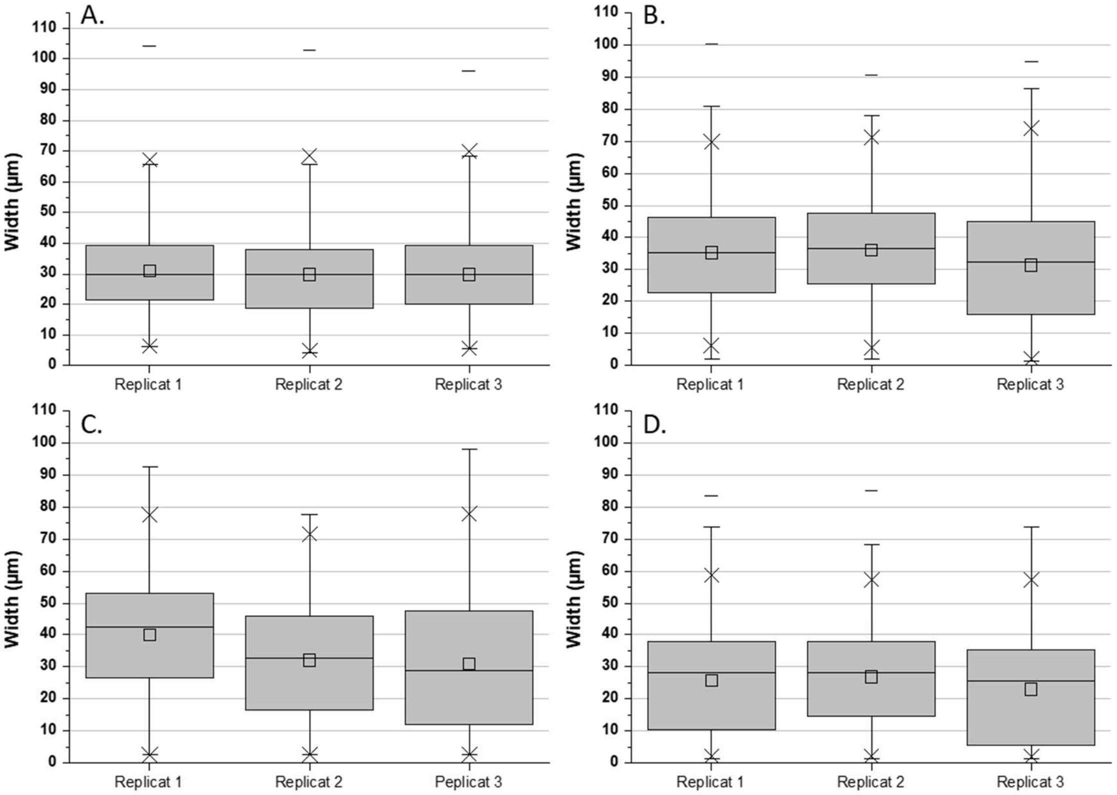

E.

	PE					PP				
	Replicat 1	Replicat 2	Replicat 3	Mean	SD	Replicat 1	Replicat 2	Replicat 3	Mean	SD
N	5092,00	5381,00	5591,00	5354,67	250,54	1094,00	1203,00	1421,00	1239,33	166,50
mean_ Aspect Ratio	0,56	0,56	0,55	0,55	0,00	0,58	0,60	0,64	0,61	0,03
mean_ Length (µm)	56,91	56,49	55,68	56,36	0,62	64,91	63,84	54,46	61,07	5,75
mean_ Width (µm)	28,68	28,33	27,69	28,23	0,50	35,27	36,06	31,46	34,26	2,46
median_ Aspect Ratio	0,55	0,55	0,54	0,55	0,01	0,59	0,62	0,64	0,62	0,03
median_ Length (µm)	55,97	57,35	55,97	56,43	0,80	62,88	62,88	57,35	61,04	3,19
median_ Width (µm)	28,33	28,33	28,33	28,33	0,00	35,24	36,62	32,48	34,78	2,11

	PET					PVC				
	Replicat 1	Replicat 2	Replicat 3	Mean	SD	Replicat 1	Replicat 2	Replicat 3	Mean	SD
N	946,00	618,00	815,00	793,00	165,10	1542,00	1486,00	1762,00	1596,67	145,89
mean_ Aspect Ratio	0,64	0,63	0,62	0,63	0,01	0,67	0,65	0,66	0,66	0,01
mean_ Length (µm)	63,90	51,63	51,08	55,54	7,25	42,58	45,03	39,42	42,34	2,81
mean_ Width (µm)	40,00	32,07	30,90	34,32	4,95	25,71	26,84	23,12	25,22	1,91
median_ Aspect Ratio	0,65	0,64	0,62	0,64	0,02	0,69	0,67	0,69	0,68	0,01
median_ Length (µm)	65,21	53,23	50,35	56,26	7,88	47,68	47,68	44,91	46,76	1,60
median_ Width (µm)	42,40	32,63	28,88	34,64	6,98	28,33	28,33	25,57	27,41	1,59

Figure S2. Width size distribution of (A) PE, (B) PP, (C) PET, and (D) PVC microparticles for fraction F5 triplicates ($25\ \mu m > F5 > 50 \mu m$) as measured by FlowCam and (E) their associated morphological statistics.

Table S1. 27 optimal experimental design used in the study.

Flow rate(µL/min)	Frequency (MHz)	Experimental mean speed (µm/s)	Predicted mean speed (µm/s)
2	1	31	26
2	1	31	31
2	5	28	28
2	10	28	30
2	15	30	31
2	15	30	31
3	1	46	44
3	5	42	43
3	10	47	44
3	15	42	45
4	1	60	62
4	5	57	60
4	10	57	59
4	15	58	61
5	1	70	72
5	1	75	76
5	5	80	76
5	10	78	76
5	15	83	79
5	15	82	77
6	1	84	88
6	1	90	89
6	5	90	90
6	10	87	88
6	10	90	91
6	15	90	92
6	15	92	90

Table S2. Percentage of particles for each polymer type (PS microbeads, PE fragments, and PP fragments) passing through the funnel stem, with (5MHz and 20 Vpp) and without the effect of DEP, based on an initial injection of 100 particles

Polymers	With DEP	Without DEP
PS	100 %	6 %
PE	100 %	7 %
PP	87 %	9 %

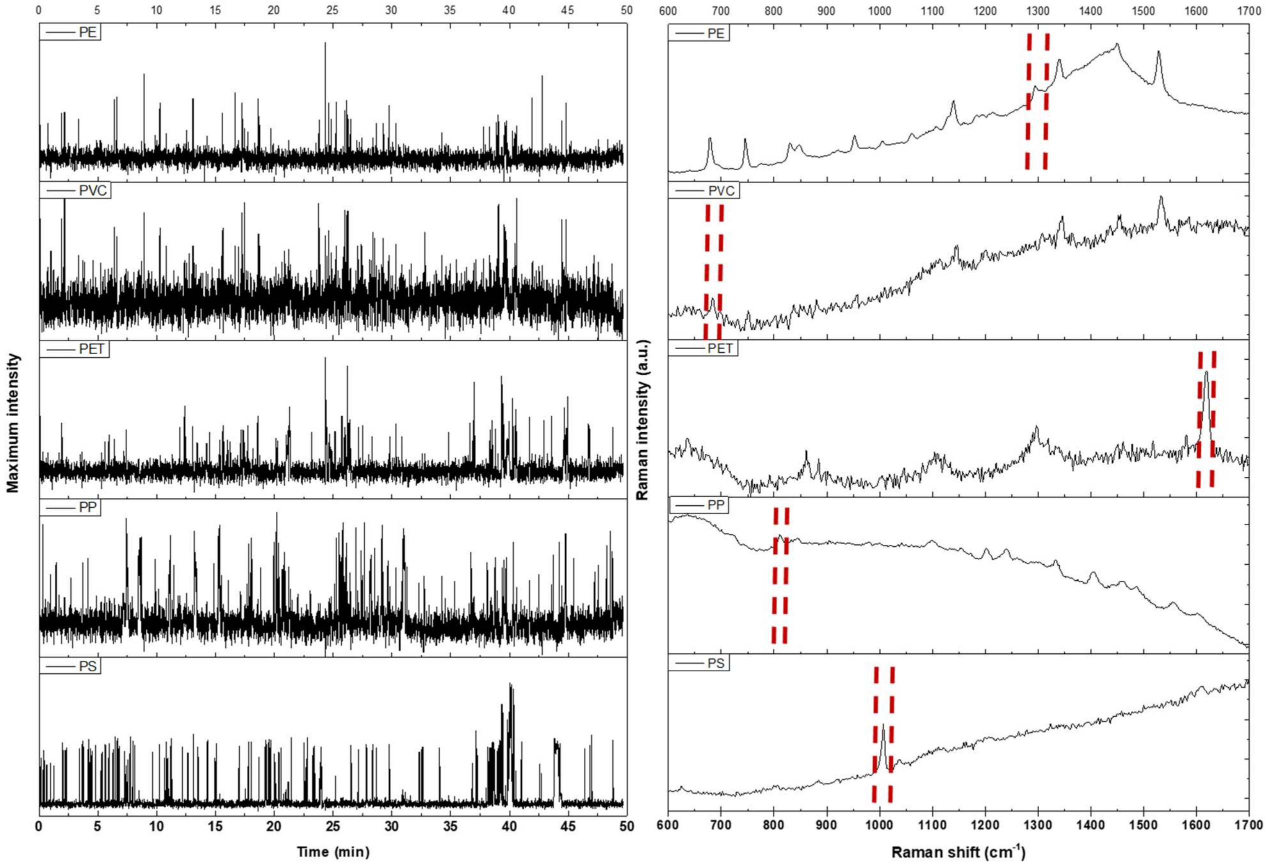

Fig. S3. Discrimination of PS, PP, PET, PVC, and PE by monitoring the maximum intensity within their characteristic Raman spectral ranges (980–1020 cm^{-1}; 814–847 cm^{-1}; 1600–1650 cm^{-1}; 670–720 cm^{-1}; and 1277–1317 cm^{-1}, respectively), as highlighted on the raw Raman spectra (right, red line) over time.

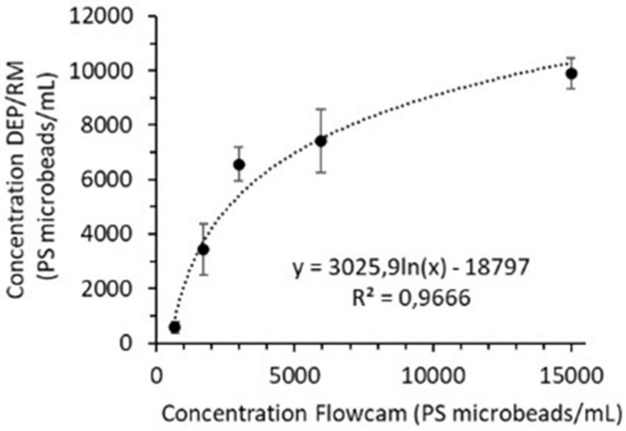

Figure S4. *Comparison plot of the concentrations obtained via DEP/RM coupling and via FlowCam (n=3) for PS microbeads.*
