# Supplementary material for: In-flow single particle detection of sub-100 micron microplastics
Source: RSC Adv. 2025 Sep 11;15(40):33139–50. doi: 10.1039/d5ra04700e (PMC12424377; doi:10.1039/d5ra04700e)
Supplement: RA-015-D5RA04700E-s002 [file RA-015-D5RA04700E-s002.pdf]

This journal is © The Royal Society of Chemistry 2025

Video analysis of PS particle via TrackMate plugin on Fiji

Raw video

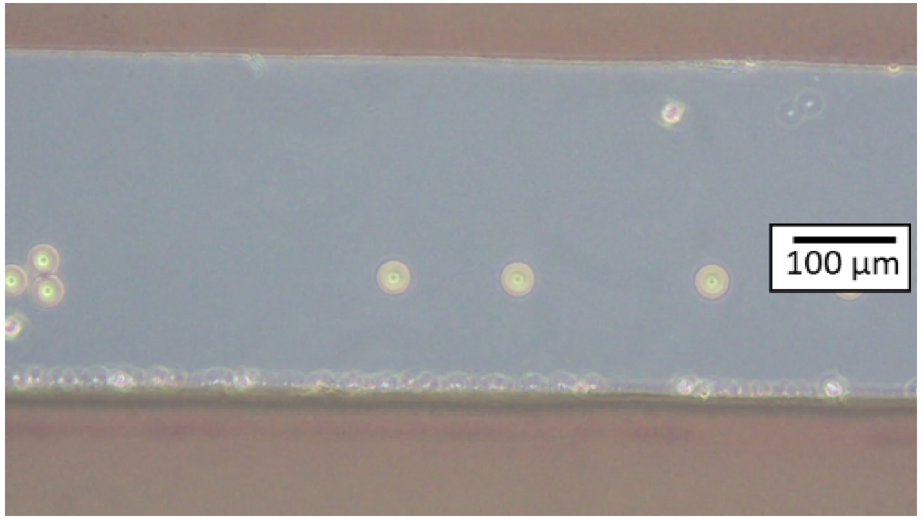

Treated video

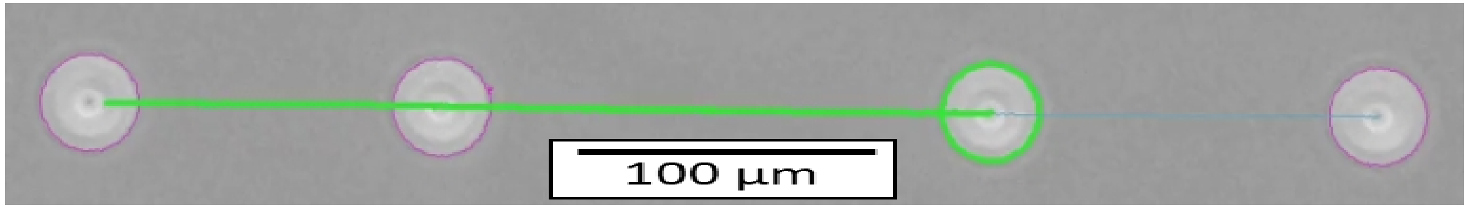

Generated data

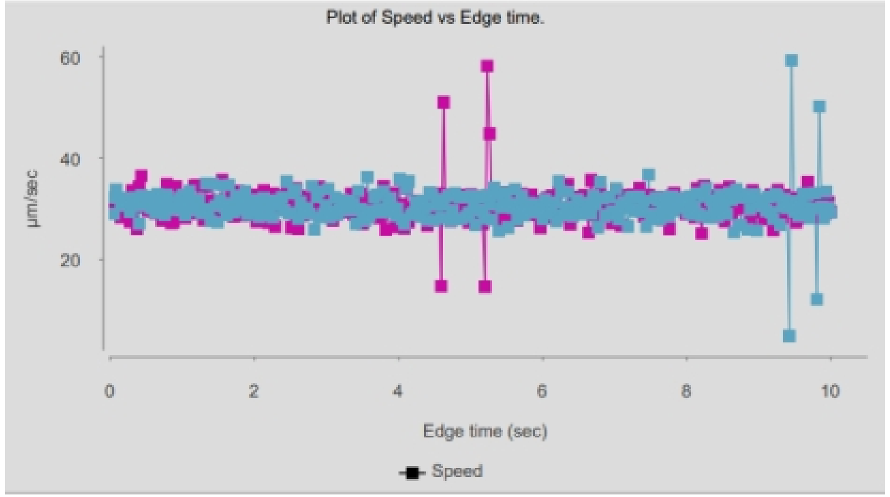

+ Mean speed, etc.

Video analysis of PE particle via TrackMate plugin on Fiji

Raw video

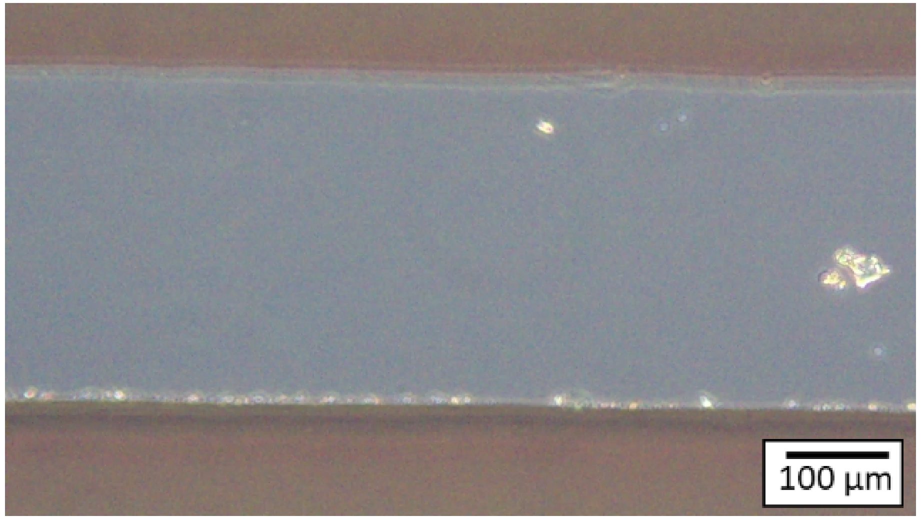

Treated video

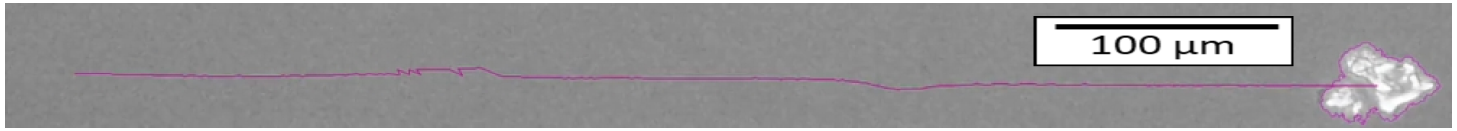

Generated data

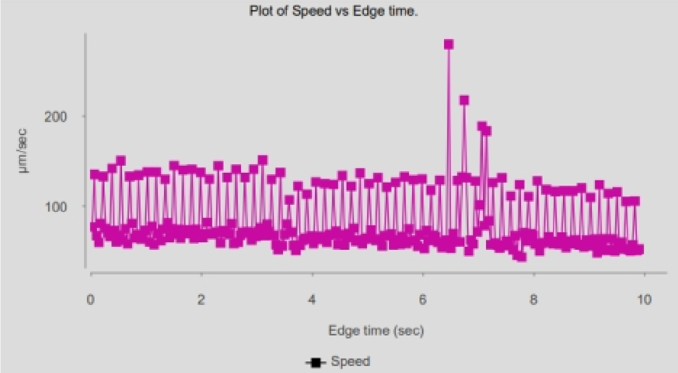

\+ Mean speed, etc.

Video analysis of PP particle via TrackMate plugin on Fiji

Raw video

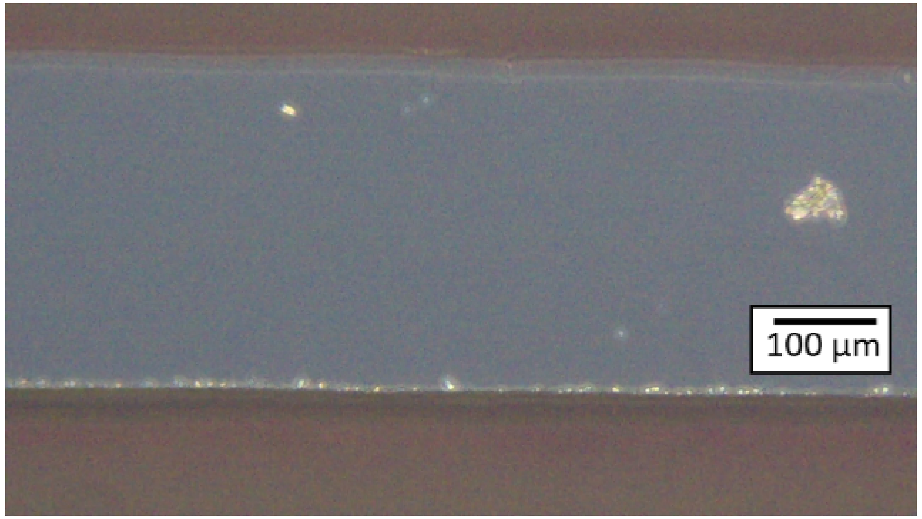

Treated video

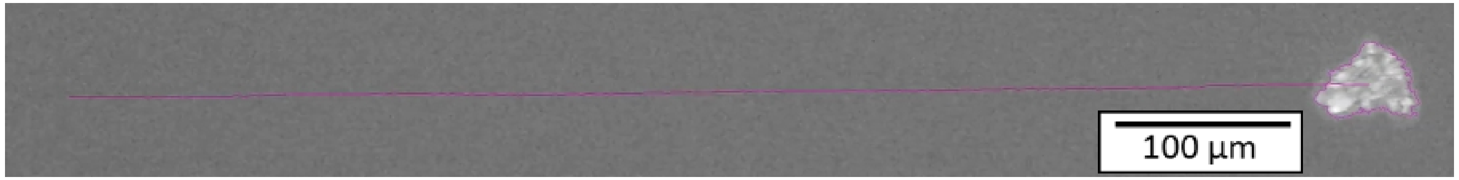

Generated data

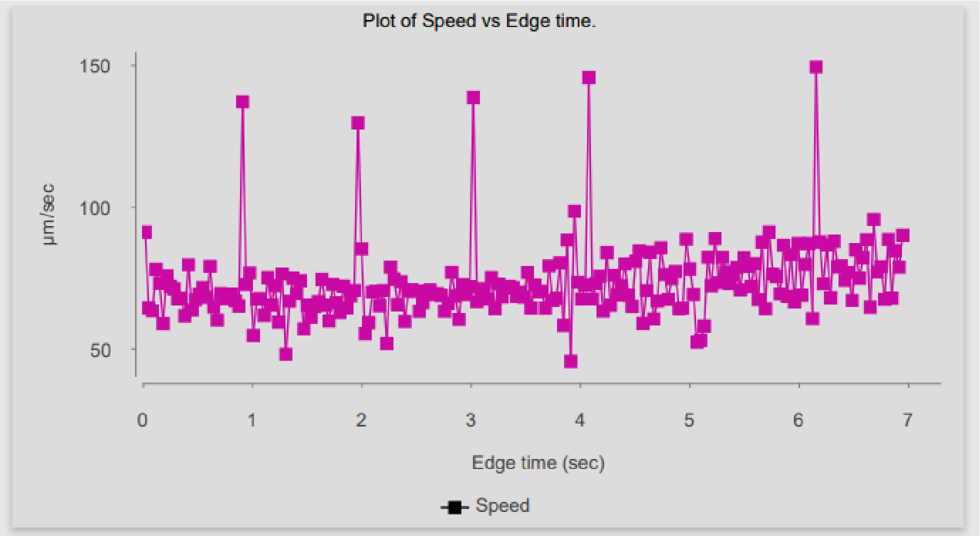

+ Mean speed, etc.
